# Submucosal gastric heterotopia presenting as an upper esophageal nodule

**DOI:** 10.1093/jscr/rjab251

**Published:** 2021-06-22

**Authors:** Jillian C Dawley, Hemanth K Gavini, Belinda L Sun

**Affiliations:** Department of Pathology, Banner-University Medical Center, University of Arizona, Tucson, AZ 85721, USA; Department of Medicine, Banner-University Medical Center, University of Arizona, Tucson, AZ 85721, USA; Department of Pathology, Banner-University Medical Center, University of Arizona, Tucson, AZ 85721, USA

## Abstract

Esophageal gastric heterotopia (GH), the presence of differentiated gastric tissue in the esophagus, is estimated in up to 14% of populations worldwide and has always been reported on the surface of the esophagus, where it is also known as inlet patch. However, submucosal GH, in any tissue, is a rare finding. We report the case of a 50 year-old male presenting with chronic cough, heartburn and raspy vocalizations. Endoscopic examination showed a single 7 mm esophageal nodule, 20 cm from the incisors, interpreted as a submucosal mass. Pathologic examination of the endoscopically excised nodule showed well-differentiated gastric mucosa within the submucosa underneath the overlying squamous mucosa, consistent with submucosal GH. This case raises the awareness of an atypical presentation and location of GH seen as a submucosal mass on endoscopy.

## INTRODUCTION

Gastric heterotopia (GH) is defined as the presence of differentiated gastric mucosa (GH)-type tissue located outside the stomach. GH commonly occurs in the proximal esophagus and rarely in the distal esophagus, where it must be differentiated from Barrett’s esophagus. In the cervical esophagus, GH exclusively occurs at the level of or just distal to the upper esophageal sphincter [1]. GH has also been described in the small intestine, rectum and biliary tree, among other sites [2]. Esophageal GH has always been reported on the mucosal surface; to our knowledge, submucosal GH in the esophagus has not been described. Here, we report a case of submucosal GH that presented as a proximal esophageal nodule in a 50 year-old male patient.

## CASE REPORT

### Clinical history

A 50 year-old male with a history of chronic gastroesophageal reflux disease (GERD) and cough was referred to university hospital for upper endoscopic ultrasonography (EUS) to evaluate a submucosal mass. He had intermittent postprandial acid reflux attacks 3 years prior with increased frequency over the next 18 months. He also complained of intermittent, postprandial dry cough and raspy vocalizations. He had an episode of chest pain leading to an emergency visit to an outside hospital, at which time cardiac and pulmonary involvement was ruled out. Physical examination was only notable for hypertension. His symptoms were improved following 2 weeks of proton pump inhibitor treatment (PPI) and a GERD diet.

A follow-up esophagogastroduodenoscopy 1 year ago at the outside hospital revealed a submucosal mass, 20 cm from the incisors, as well as moderate reflux esophagitis and antral chronic gastritis without *Helicobacter pylori*. He was then referred to our hospital to evaluate the submucosal mass. At the time of examination here, he reported that PPIs were no longer improving his symptoms.

### Endoscopic and ultrasonographic findings

**Figure 1:**
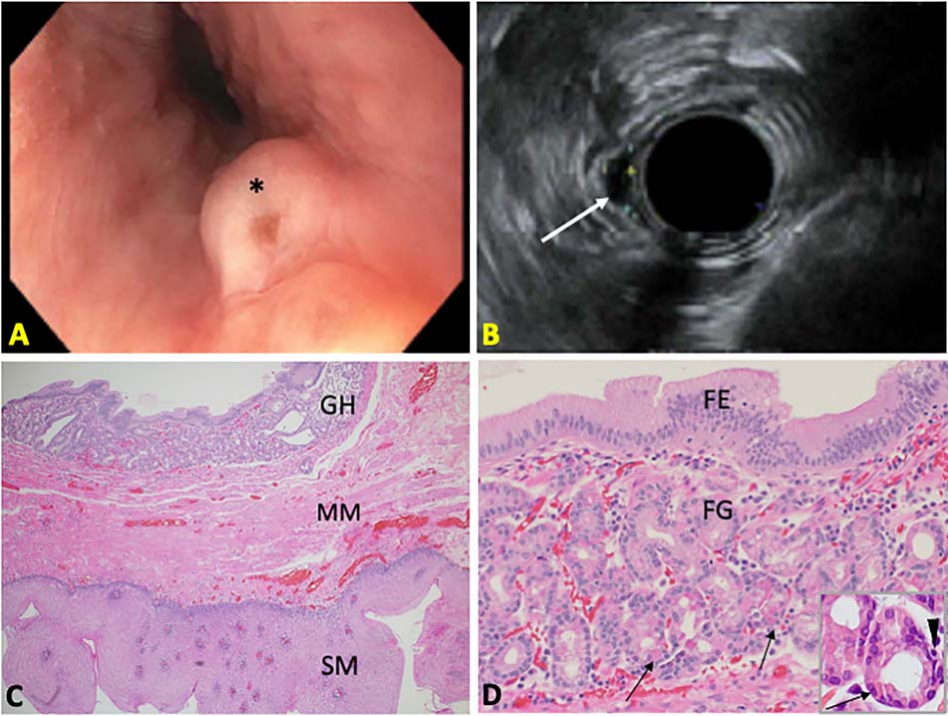
(**A**) Endoscopic image of the esophageal nodule (^*^). (**B**) Endosonographic image showing a hypoechoic, oval subepithelial lesion (arrow). (**C**) Submucosal heterotopic GH beneath the MM of esophageal SM (H&E 20X). (**D**) Superficial foveolar epithelium (FE) and deep fundic glands (FG) with parietal cells (arrows) and neuroendocrine cells (arrowhead) (H&E 50X; inset: 100X).

Upper EUS demonstrated a 0.7 cm submucosal nodule (Fig. 1A) in the proximal esophagus, 20 cm from the incisors and just distal to the upper esophageal sphincter. Other findings included gastroesophageal flap valve, Hill Grade IV; gastric fluid pH of 2; mildly erythematous mucosa in the gastric antrum and three superficial duodenal ulcers in the duodenal bulb.

Endosonographic evaluation of the esophageal nodule showed a hypoechoic oval intramural (subepithelial) lesion with well-defined borders and apparent origin within the deep mucosa (Fig. 1B). A preliminary diagnosis of stromal cell neoplasm was made at the time of endoscopy. Complete endoscopic resection and retrieval of the nodule were achieved for diagnostic purposes.

### Pathologic findings

Gross examination of the excised esophageal nodule showed a pink soft tissue nodule, 1.0 cm in diameter and 0.3 cm thick. Microscopic histopathologic examination showed orderly gastric epithelium and glands beneath the muscularis mucosae (MM) of the esophageal squamous mucosa (SM), consistent with submucosal GH (Fig. 1C). This GH contained superficial glands, consisting of surface foveolar cells and mucous neck cells, and deeper glands, containing parietal, chief, and neuroendocrine cells (Fig. 1D). There was no dysplasia, inflammation or *H. pylori* organisms identified.

### Follow up

There was verbal follow up with the patient 2 weeks after upper EUS. It is unclear whether the patient derived benefit from the intervention as he reported ongoing symptoms of GERD not alleviated by PPI.

## DISCUSSION

Here, we report a case of submucosal esophageal GH in a 50 year-old man presenting with complaints of heartburn, chronic cough and raspy vocalizations. The GH presented endoscopically as a nodule just distal to the upper esophageal sphincter. Histopathologic evaluation showed gastric-type mucosa without inflammation or dysplasia. To our knowledge, this is the first report of submucosal esophageal GH.

Prior case reports of esophageal GH have been mucosal lesions where the GH interrupts the esophageal squamous epithelium or is located just beneath it [3]. There is one report of submucosal gastric glands associated with an inclusion cyst in the distal esophagus [4]. Descriptions of submucosal GH in locations other than the esophagus are also sparse. Yamagiwa *et al*. [5] described submucosal GH in resected stomachs. Isolated submucosal GH causing recurrent intussusception has been described in the small intestine [2].

The earliest description of GH was a report of esophageal heterotopic GH discovered on postmortem examination by Schmidt in 1805. Islands of GH in the mucosa of the cervical esophagus are referred to as inlet patches. The prevalence of inlet patches ranges from 0.18 to 14%, though autopsy reports show an incidence of up to 70% [3, 6]. Additionally, inlet patches are observed in the pediatric population with a prevalence of up to 5.9% [7]. The endoscopic detection of esophageal GH is dependent on the endoscopist’s awareness of GH [8].

GH is widely considered to be congenital in origin, representing remnants of the columnar lining of the fetal esophagus. The embryonic esophagus, initially lined by ciliated columnar epithelium, is later replaced by non-keratinized squamous epithelium, beginning at the mid-esophagus and proceeding proximally and distally. Failure of this squamous epithelialization is thought to be the cause of GH. In contrast, some have suggested GH as an acquired condition, representing healing from chronic injury, similar to the pathogenesis of Barrett’s esophagus. Support for the acquired hypothesis includes the similar expression of cytokeratin and mucin protein in patients with inlet patches and Barrett’s esophagus.

GH is largely asymptomatic and discovered incidentally. Some symptoms include heartburn, chronic cough and hoarseness, which may be attributed to the potential of inlet patches produce acid, but mucous production has also been implicated [9, 10]. Complications of esophageal GH include laryngitis, esophagitis, esophageal web, stricture, ulcer, perforation and fistula [10]. Dysplasia and adenocarcinoma in association with GH have been reported but the incidence is rare [3]. In the present case, this patient had a long history of heartburn and cough, and a more recent history of raspy vocalizations. Histologic examination of this patient’s GH showed differentiated parietal cells, which may have the function of acid production and contribute to this patient’s symptoms. However, the clinical significance of this GH remains in question.
